# The Role of Reactive Oxygen Species in Colorectal Cancer Initiation and Progression: Perspectives on Theranostic Approaches

**DOI:** 10.3390/cancers17050752

**Published:** 2025-02-22

**Authors:** Teresa Catalano, Federico Selvaggi, Roberto Cotellese, Gitana Maria Aceto

**Affiliations:** 1 Department of Clinical and Experimental Medicine, University of Messina, Via Consolare Valeria, 98125 Messina, Italy; 2 Villa Serena Foundation for Research, 65013 Città Sant’Angelo, Italy; fedeselvaggi@hotmail.com (F.S.); roberto.cotellese@unich.it (R.C.); 3 Department of Sciences, University “G. d’Annunzio” Chieti-Pescara, Via dei Vestini 31, 66100 Chieti, Italy

**Keywords:** ROS, H_2_O_2_, CRC progression, biomarkers, theranostic, surgery, CRC therapy

## Abstract

Altered levels of reactive oxygen species (ROS) are recognized as one of the key factors in mediating tumor cell survival in the tissue microenvironment, where they play a role in the initiation, progression and recurrence/relapse of colorectal cancer (CRC). Tumor cells can adapt to oxidative stress (OS) using genetic or metabolic reprogramming in the long or short term. In addition, tumor cells defend themselves through positive regulation of antioxidant molecules, enhancing ROS-driven proliferation. Balanced oxidative eustress levels can influence chemotherapy resistance, allowing tumor cells to survive treatment. Secondary effects of chemotherapy include increased ROS production and redox stress, which can kill cancer cells and eliminate drug resistance. Anticancer treatments based on manipulating ROS levels could represent the gold standard in CRC therapy. Therefore, exploring the modulation of the response to OS in deregulated signaling pathways may lead to the development of new personalized CRC treatments to overcome therapy resistance. In this review, we explore the role of ROS in the initiation and progression of CRC and their diagnostic implications as biomarkers of disease. Furthermore, we focused on the involvement of ROS in different CRC therapeutic options, such as surgery, radiotherapy, theranostic imaging, chemotherapy and immunotherapy and other precision medicine approaches.

## Simple Summary

Oxidative stress plays a key role in mediating cancer cell survival in colorectal cancer (CRC) initiation and progression. ROS production regulates and activates growth, repair and death pathways. Cancer cells adapt to oxidative stress in the long or short term by genetic or metabolic reprogramming. Therefore, exploring the modulation of the oxidative stress response in deregulated pathways may lead to the promotion of new personalized treatments for CRC to overcome resistance to therapies. Based on the current literature, we proposed an update of the molecular mechanisms underlying ROS-driven proliferation, focusing on the involvement of ROS in CRC therapeutic options such as surgery, theranostic imaging and precision medicine approaches.

## 1. Introduction

**Figure 1:**
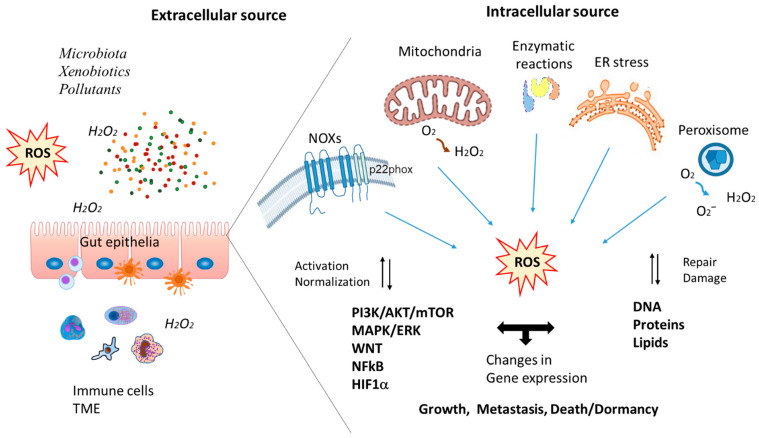
Schematic representation of intracellular and extracellular stimuli that induce ROS in gut epithelia. Extracellular sources of ROS include environmental factors such as microbiota products, xenobiotics and pollutants. Intracellular sources of ROS are mainly from mitochondria, endoplasmic reticulum (ER) and peroxisomes [15]. NADPH oxidases (NOXs) form multimeric complexes that generate superoxide or H_2_O_2_. NADPH oxidases, present in the gut as NOX1 and NOX2, heterodimerize with p22phox to convert molecular oxygen to superoxide [2]. ROS production regulates and activates growth, repair and death pathways through changes in gene expression (illustration created using some icons from BioRender.com, accessed on 8 January 2025).

The underlying mechanisms by which oxidative stress (OS) mediates cancer initiation, progression and treatment resistance in colorectal cancer (CRC) are not fully understood. In a living organism, OS is a pathophysiological condition caused by an imbalance between the production of reactive oxygen species (ROS) and their elimination by the antioxidant system [1]. In the gut, ROS can be released by the immune system, epithelium and microbiota [2] (Figure 1). ROS are generated during normal physiological processes of growth, renewal and adaptation of cells and tissues and are released as metabolic by-products of aerobic respiration, some enzymatic processes or immune responses [3,4]. Under physiological conditions, the appropriate redox state determines the homeostasis of the intestinal epithelium by regulating signaling pathways responsible for the proliferation, crypt-villus migration and differentiation of intestinal stem cells [5]. Two important signaling pathways of enterocyte self-renewal, proliferation, migration and differentiation, Wingless/It(Wnt)/β-catenin and Notch, are redox-sensitive and can be modulated by the activity of nicotinamide adenine dinucleotide phosphate (NADPH) oxidases [3,6]. Indeed, NADPH oxidase 1 (NOX1), an ROS-generating oxidase, is highly expressed in colon epithelial cells where it controls the rate of proliferation and post-mitotic differentiation by coordinately modulating the phosphoinositide-3-kinase (PI3K)/protein kinase B (AKT)/Wnt/β-catenin and Notch1 pathways [2,7]. Major sites of ROS production are mitochondria, membrane-bound NOXs and peroxisomes during the process of respiration [8]. Hydroxyl radical and singlet oxygen are the most reactive forms of ROS; they rapidly oxidize all biological molecules, especially unsaturated fats, proteins and nucleic acids (Figure 1). Furthermore, NOX1-derived hydrogen peroxide (H_2_O_2_) can regulate the growth and survival of bacteria near the mucosal surface during intestinal homeostasis [8]. Dysregulated production of ROS, including H_2_O_2_, in the colon epithelium is associated with the presence of microbial products from the gut microbiota, the innate immune system defense strategy and the effects of exposure to xenobiotic chemicals (i.e., chemotherapeutic agents, environmental organic pollutants) [2,9]. The intestinal epithelium performs essential functions for the organism, both as a semi-permeable barrier for nutrient absorption in a symbiotic relationship with the intestinal microbiota and as an immune defense of the organism itself [10]. Absorptive enterocytes are labile cells that must ensure the integrity of the barrier through tight and adherent junctions, so worn cells are regularly replaced thanks to the activity of adult intestinal stem cells (ISCs), which can ensure the replacement of all types of intestinal epithelial cells [10,11]. Evolutionarily conserved molecular signals allow the maintenance of intestinal tissue homeostasis through signaling pathways such as Wnt, epidermal growth factor receptor (EGFR), hippo, Notch, hedgehog and bone morphogenetic protein (BMP) to control the proliferative activity and fate of ISCs [5,11,12]. Efficient control of the molecular crosstalk between these signaling pathways may prevent excessive ISC proliferation and, thus, tissue hyperplasia and tumor initiation [12,13,14]. OS qualitatively and quantitatively regulates many of these signaling pathways in cellular compartments. Cellular redox reactions, housed in the mitochondria, peroxisomes and endoplasmic reticulum (ER), generate H_2_O_2_ and other ROS [15,16]. Common ROS species in gut epithelia include hydroxyl radicals (•OH), superoxide anion (O2•–) and H_2_O_2_ [17] (see Figure 1). Proteins, lipids and nucleic acids can be indiscriminately oxidized and damaged by excessive production of these reactive species. In particular, H_2_O_2_ reacts with Fe^2+^ (Fenton reaction) or superoxide anion (Haber–Weiss reaction) to form the hydroxyl radical, the production of which is increased under pathological conditions [1]. Therefore, iron accumulation may also contribute to colon tumorigenesis. Lipid peroxidation appears to be increased in CRC compared to normal mucosa [18]. Lipid peroxidation promotes free radical reactions and facilitates the formation of DNA adducts, which contribute to tumorigenesis [19]. OS is normally balanced by the presence of antioxidants, such as superoxide dismutase (SOD), glutathione (GSH), glutathione peroxidase (GPx), catalase and thioredoxin, which neutralize reactive species and maintain cellular homeostasis [20]. However, when confined in space and time, oxidation can act as a signaling event [13]. Typically, cancer cells generate higher basal levels of ROS compared to normal cells as a result of deregulation imbalance [18]. Elevated H_2_O_2_ levels are a feature of the tumor microenvironment that differs from normal tissues [21]. OS plays a key role in mediating cancer cell survival in CRC progression [22]. The beneficial or detrimental effects of ROS on the regulation of various cellular processes depend on their levels. Thus, ROS exhibits a multifaceted (dual) role in cancer cell survival and functions through pro-tumorigenic or anti-tumorigenic activities depending on their intracellular concentration, the origin of the tumor cells and the activated pathways [23,24,25]. Colorectal carcinogenesis and progression occur through a multi-step process, most often starting from an adenoma that has the potential to progress to carcinoma through deregulation of Wnt/β-catenin/adenomatous polyposis coli (APC) signaling with subsequent accumulation of epigenetic alterations and somatic mutations and driven by interactions with the metabolic and immune status of the tissue microenvironment (TME) [12,14,26]. Cells can adapt to OS in the long term by genetic reprogramming and/or temporarily by metabolic reprogramming, which provides tumors with energy and intermediate metabolites to sustain the rapid proliferation of cancer cells [27,28]. The metabolic pathways utilized by tumor cells are related to glucose, amino acid and lipid metabolism and are associated with their fate and phenotype [29]. These metabolic interactions could influence the initiation and progression of CRC and its response to chemotherapy. Metabolic reprogramming in CRC is also associated with the mutational status of Wnt/β-catenin pathway components and other genetic drivers, such as KRAS, BRAF, the tumor suppressor p53, EGFR and transforming growth factor-β (TGF-β), which promote tumor initiation and progression [29,30,31,32,33]. Metabolic reprogramming in the metastatic progression of CRC may be regulated by OS in the TME. Indeed, it has been observed that CRC cells derived from primary tumors or metastases undergo differential modulation of metabolic capacities in the presence of acute oxidative H_2_O_2_ distress or eustress, such that they can utilize canonical and non-canonical Wnt/β-catenin/APC molecular pathways also in crosstalk with the c-Jun N-terminal kinase (JNK) pathway [34,35]. Investigations into the dynamic events that may influence cancer initiation and progression have revealed the existence of a crosstalk between the dysregulated intracellular production of ROS by tumor cells and the extracellular ROS excreted by the cells constituting the TME, such as macrophages, tumor-associated macrophages (TAMs), neutrophils, tumor-associated neutrophils (TANs), activated fibroblasts, such as cancer-associated fibroblasts (CAFs) and stromal and epithelial cells [36,37]. Indeed, ROS generated by the cells of the TME modify the cancer niche and exert their action on numerous cell types and matrix components [38]. The interplay between the cancer niche and tumor cells is important in promoting cancer growth, metastasis and treatment response. Intrinsic and extrinsic factors regulate ROS activity on distinct cellular functions, including self-renewal, tissue homeostasis and repair, dormancy, senescence, death and the anticancer immune response through the activation of inflammatory and immune cells. This occurs despite the fact that high levels of ROS in the TME inhibit T-cell cytotoxicity, leading to tumor invasion and treatment resistance during cancer progression [12,14,25,37]. Furthermore, numerous cancer processes are redox-sensitive, including cell cycle progression and proliferation, motility, survival, apoptosis, metabolism, cell-to-cell adhesion, angiogenesis, tumor stemness and therapeutic resistance via a variety of mechanisms [39,40]. In cancers such as CRC, redox modulation of responses in the TME could be very useful for increasing efficacy and therapeutic success [38]. In this review, we explore the role of ROS in mediating CRC initiation and progression and their diagnostic implications as biomarkers of the disease. We also evaluate the therapeutic potential of ROS in CRC.

## 2. Reactive Oxygen Species Play a Role in Mediating CRC Initiation

Exposure of normal cells to external inducers of moderate or high levels of oxygen radicals determines cellular changes and contributes to transformation into malignant cells by inflammation and DNA damage [12]. This occurs in response to environmental pollutants, chronic infections, ultra-processed food, alcohol intake, tobacco consumption, drugs and xenobiotics, toxic compounds or ionizing and ultraviolet radiation [41,42]. Gut microbiota plays a key role in CRC pathogenesis by influencing initiation, development, and progression. Alterations in gut microbiota homeostasis support CRC-promoting changes in the gut microenvironment [43]. Microbial dysbiosis induced by some bacterial species, such as *Fusobacterium nucleatum* and *pks+ Escherichia coli*, causes inflammation and generation of ROS in the intestinal mucosa, directly modulating the neoplastic transformation of intestinal epithelial cells or indirectly through the interaction with the host immune system [43,44]. *Actinomyces odontolyticus* is a bacterium present in the gut in the initial stages of CRC. It produces membrane vesicles (MVs) rich in lipoteichoic acid, which are internalized into colonic epithelial cells and generate mitochondrial dysfunction. *A. odontolyticus* induces the nuclear factor-kappa B (NF-κB) pathway, resulting in the excessive production of ROS in colonic epithelial cells, leading to DNA damage, as well as intestinal dysplasia, leading to the initiation of CRC [45]. Conversely, some bacteria in the gut microbiota protect the function of the intestinal epithelial barrier against CRC by producing metabolites or regulating inflammation and the immune system [43].

Compared to normal cells, tumor cells generate higher levels of ROS as a consequence of deregulated activation of oncogenic signaling pathways due to genetic, epigenetic and metabolic changes, such as elevated aerobic glycolysis, mitochondrial dysfunction and/or alterations in the tumor microenvironment [25,38,46]. Indeed, ROS display many protumorigenic properties, such as induction of cell proliferation, tumor growth, adaptation to hypoxia, inhibition of apoptosis, epithelial-to-mesenchymal transition (EMT), migration, metastasis, angiogenesis and chemoresistance [37,46,47,48]. On the other hand, CRC cells protect themselves from oxidative damage by evading apoptosis and upregulating antioxidant molecules, such as SOD, GPX, thioredoxin/thioredoxin reductase (Trx/TrxR) system and glutathione S-transferases (GSTs) [27,49].

In cancer cells, this antioxidant activity is upregulated through transcription factor nuclear factor-erythroid 2 p45-related factor 2 (NRF2) [23]. Increased levels of NRF2 lead to nuclear translocation of NF-κB and a very high production of pro-inflammatory cytokines; in contrast, the antioxidant molecules prevent the entry of NF-κB into the nucleus [50]. The most common and widely studied DNA lesion resulting from oxidative damage is caused by an ROS attack on guanine at position C8, resulting in the formation of 7,8-dihydro-8-oxoguanine (8-oxoG). In nascent DNA, 8-oxoG leads to the formation of 8oxoG:A pairs, which are relatively stable and can easily escape the corrective activity of DNA polymerase, inducing G:C to T:A transversions [51,52]. Early steps in the initiation and promotion of colorectal tumorigenesis often involve oxidative DNA damage [3]. The removal of oxidative damage lesions from DNA is realized mainly by the base excision repair (BER) pathway. Several DNA repair enzymes restore the damage induced by 8-oxodG [53]. Among them are 8-oxoguanine DNA glycosylase 1 (OGG1), MutY homolog Escherichia coli, a homolog of MYH, hMYH (MUTYH), an enzyme to repair DNA after identification and removal of 8-OHdG [53,54]. The activity of OGG1 and MUTYH enzymes is regulated by ROS [53,55]. Intracellular H_2_O_2_ can be transformed into a hydroxyl radical, leading to a global increase in 8-oxoguanine (8-oxoG), which in turn can induce oxidative DNA damage in proliferating cells and may regulate EGFR and mitogen-activated protein kinase (MAPK) signaling, which contributes to redox protein-mediated cancer progression [53,56]. During cancer initiation, genetic alterations facilitate tumor cell survival by activating antioxidant transcription factors or by increasing NADPH levels through the pentose phosphate pathway (PPP) [27]. Continuous exposure to OS also depletes the antioxidant capacity of intestinal mucosal cells, causing them to enter a phase of chronic inflammation [50].

Patients with CRC can have a deregulation of iron transportation and abnormal iron homeostasis [57]. Previous studies found an association between mutations in the *HFE* gene, responsible for the iron overload disorder hereditary hemochromatosis, and a predisposition to an increasing risk of colon cancer [58,59,60,61]. Despite this, a more recent study asserted that the hemochromatosis genotype is not associated with CRC or age at its diagnosis [62]. However, iron activates Wnt signaling in CRC cell lines harboring APC or β-catenin mutations, while in cell models containing wild-type APC and β-catenin mutation, Wnt signaling is activated by iron, which only regulates β-catenin [63]. When Fe^2+^ levels are overloaded in cells, Fe^2+^ and H_2_O_2_ undergo the Fenton reaction to form hydroxyl radicals (•OH), which oxidize polyunsaturated fatty acids (PUFAs) and lead to cell damage and rupture of cell membranes. This effect of iron increases free radical reactions and also facilitates the formation of DNA adducts that contribute to CRC tumorigenesis [57]. The impaired GSH activity, iron overload and accumulation of lipid peroxides have been proposed as the main mechanisms and features of a type of iron-dependent nonapoptotic form of regulated cell death, termed ferroptosis [64]. Iron accumulation in CRC leads to increased production of ROS, activation of oncogenes, pro-inflammatory mediators and dysbiosis that contribute to increased CRC growth [65]. Indeed, CRC is associated with modifications in gut microbiota. Iron therapy supplementation following iron deficiency due to anemia in CRC may contribute to the formation of a procarcinogenic microbiota, as it may select for pathogenic bacterial species associated with a reduction in protective and non-harmful bacteria [66]. This produces an imbalance in bacterial populations. Pathogenic bacteria can invade the intestinal wall, causing inflammation and activating carcinogenic metabolites and signaling pathways [67]. Failure of iron homeostasis induces ROS production and ferroptosis via c-Myc/NRF2-mediated signaling in CRC [68].

TP53-induced regulator of glycolysis and apoptosis (TIGAR) is downstream of p53 and exerts antioxidant activity in cells. Furthermore, TIGAR knockout increased the sensitivity of CRC cells to ferroptosis by reducing Stearoyl-CoA desaturase-1 (SCD1) expression in a redox and 5′ AMP-activated protein kinase (AMPK)-dependent manner. This suggests that targeting TIGAR to activate ferroptosis in CRC may be a therapeutic approach [69]. A direct link associates iron metabolism and the p53 signaling pathway. Indeed, heme directly binds to the p53 protein, regulating its stability [70]. Heme binding interferes with p53-DNA interactions, leading to conformational changes of p53 that are responsible for its nuclear export and cytosolic degradation [71]. Therefore, iron excess in cancer may sustain heme synthesis, affecting p53 stability and function and leading to the downregulation of p53 levels. Thus, iron metabolism may inactivate the p53 signaling pathway and induce tumorigenesis [70]. On the other hand, p53 activation decreases GSH since it reduces the cystine uptake and promotes ferroptosis through the transcription of the system xc− transporter (xCT), a cystine/glutamate antiporter that plays a critical role in the maintenance of GSH homeostasis through the regulation of the Na+-independent uptake of extracellular cystine that exchanges for intracellular glutamate [72,73]. xCT forms the heterodimeric amino acid transport system xc- in association with the chaperone protein 4F2 heavy chain. ROS upregulate xCT through the translocation of NRF2 into the nucleus and its binding to the *SLC7A11* gene promoter, leading to xCT protein production. xCT expression is regulated by wild-type and mutant p53. Mutant p53 protein suppresses the expression of the components of the xCT system SLC7A11 by binding to NRF2. This decreases GSH synthesis, exposing tumor cells to OS [74].

DNA genotoxic alterations can include base modifications, single and double-strand breaks, deletions, insertions, chromosomal mutant-p53 translocations, rearrangement of sequence, miscoding lesions, gene amplification, upregulation of oncogenes and inactivation of tumor suppressor genes [50,75]. In particular, double-strand DNA breaks can alter the levels or functions of ‘modifier’ proteins, thereby determining cancer progression [25]. Intracellular redox imbalance also induces aberrant activation of pathways such as Wnt/β-catenin, PI3K/AKT and Janus kinase (JAK)–signal transducer of activators of transcription (STAT) signaling pathways [76,77]. Wnt/β-catenin or canonical pathway and non-canonical planar cell polarity (Wnt-PCP) and Wnt-Ca^2+^ signaling pathways are involved in cancer cell proliferation, stemness, apoptosis, metabolism, inflammation, immunity, microenvironment regulation, resistance, migration, invasion and metastasis. Wnt signaling is a known driver of CRC. APC is involved in regulating the homeostasis of colon epithelium renewal, cell cycle progression, differentiation, migration and apoptosis on the crypt–villus axis [5]. APC and KRAS mutations play a critical role in the development and progression of CRC [78,79]. APC is a key regulator of Wnt signaling, and approximately 90% of human CRC cases are associated with defects in the Wnt pathway. Alterations in canonical APC function lead to aberrant stabilization of β-catenin, a critical event in the initiation of CRC. Specifically, loss of APC canonical function induces alterations in intestinal differentiation, and the transcriptional corepressor C-terminal binding protein-1 (CtBP1) influences the adenoma initiation [80,81]. As a result of APC mutations, Rac Family Small GTPase 1 (Rac1) is involved in the expansion of LGR5 intestinal stem cell signature and in the increased proliferation and transformation of progenitor cells. ROS production and activation of NF-κB pathways triggered by Rac1 are essential in inflammation and initiation of CRC [82,83]. Activation of the Wnt signaling can be the consequence of Rac1 induction by the ligand–receptor complex to promote ROS production, which then oxidizes the oxidoreductase enzyme nucleoredoxin (NRX). This contributes to cellular redox homeostasis by regulating the WNT/β-catenin pathway via interaction with Dishevelled protein 1 (Dvl). Indeed, the dissociation of Dv1 from NRX results in a lack of degradation of β-catenin, with activation of target genes involved in carcinogenesis [76]. The Wnt/β-catenin pathway can be regulated through phosphatase and tensin homolog (PTEN) oxidation by NOX1 [22]. The Wnt/β-catenin and RAS-extracellular signal-regulated kinase (ERK) pathways interact in tumorigenesis. Multiple mutations of both signaling pathways significantly increase cooperative tumorigenesis, including initiation and progression [83,84]. An advanced adenoma arises from an adenoma as a consequence of an oncogenic KRAS driver mutation. Ras mutations result in the aberrant activation of the Raf-Mitogen-activated protein kinase kinase–MAPKK (MEK)-ERK and PI3K-Akt signaling pathways [83]. Ras proto-oncogene induces superoxide production mediated by the upregulation of NOX1 through the activation of the MAPK pathway [85]. ROS promote cellular transformation if cells achieve escape from cell death. Oncogenic KRAS provides cell evasion of extrinsic apoptosis through MAPK, PI3K and Rac1 signaling pathways [86]. Furthermore, oncogenic Ras triggers an antioxidant program to support tumorigenesis at tumor initiation, with redox adaptation, transformation, proliferation and resistance to apoptosis [87]. In tumor cells, positive regulation of antioxidant molecules enhances ROS-driven pro-proliferative signaling pathways by reducing the activation of senescence, apoptosis and ferroptosis [27,88]. Indeed, early genetic alterations in KRAS activate cellular transformation with the activation of antioxidant mechanisms, such as the light-chain subunit of the system xc− transporter (xCT), a cystine/glutamate antiporter, NRF2 and gamma-glutamyltransferase 2 (GGT2). This results in changes in intracellular metabolism, such as regulation of GSH and NADPH production via the tricarboxylic acid cycle (TCA) through the glutamine metabolism mediated by aspartate aminotransferase/glutamic-oxaloacetic transaminase 1 (GOT1), or production of NADPH via fatty acid oxidation mediated by acyl-coenzyme A (CoA) synthetase long-chain family member 3 (ACSL3). Subsequently, Ras activates pro-oxidant pathways, such as NOX and COX-2, resulting in additional mutations, which stimulate tumor progression [87]. High levels of ROS could activate PI3K/AKT signaling pathways through oxidation of PTEN cys124 and induce colorectal carcinogenesis [22]. Since EGFR is involved in the deregulation of the PI3K signaling pathway, the redox change of EGFR may induce the activation of the PI3K pathway [76]. ROS can activate the JAK/STAT signaling pathway in CRC carcinogenesis, overexpression of cyclin D1 and inhibition of CRC cell apoptosis [76]. Oxidative modification of STAT3 cys253 can produce dimerization of STAT3, which induces its nuclear translocation [22]. ROS can directly oxidize components of the MAPK cascades. H_2_O_2_ can decrease the phosphorylation level of p38, ERK1/2 and JNK through inhibition of MEK1/2 [76] (Table 1). Phosphorylation of the ERK1/2 pathway is reduced in the early steps of colon tumorigenesis, although it increases in advanced metastatic CRC and appears to be associated with H_2_O_2_. Therefore, ERK2 shows a pro-growth role, whereas ERK1 has a regulatory role in colon carcinogenesis [88].

## 3. Reactive Oxygen Species Play a Role in Mediating CRC Progression

**Figure 2:**
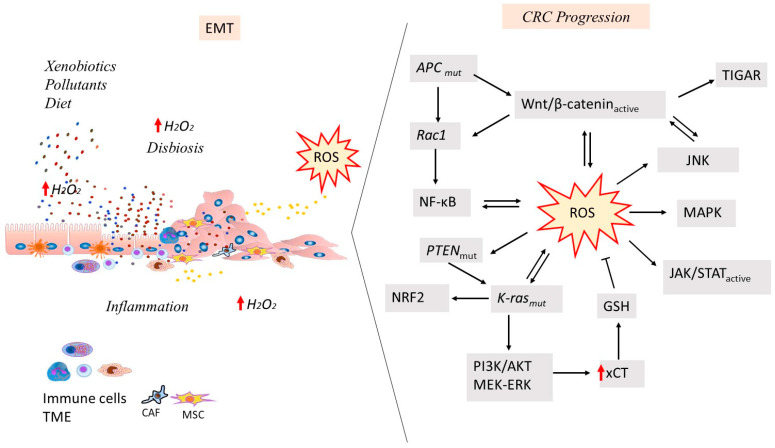
Schematic representation of intracellular and extracellular stimuli that induce ROS and CRC progression. Aberrant activation of pathways by ROS deregulation.

As sporadic CRC progresses, genetic instability can determine a chromosomal instability (CIN) phenotype in 85% of cases or a hypermutated microsatellite instability (MSI) phenotype in approximately 15% of cases. From adenoma to carcinoma, a large number of recurrent driver mutations in the *APC*, *KRAS*, *SMAD4* and *TP53* genes tend to accumulate in tumor tissue [84,89]. In the TME, altered OS response and metabolic reprogramming contribute to CRC cell survival and proliferation by increasing oxygen-independent glycolysis (Warburg effect) and molecular signals responsible for physiological epithelial renewal, such as the Wnt/β-catenin system in the colon [34,90]. In the later stages of tumor transformation, high levels of ROS associated with oxidative DNA damage contribute to the maintenance of genomic instability in tumor cells, allowing them to survive and proliferate in the TME, promoting angiogenesis, invasion and metastasis [14] (Figure 1 and Figure 2). Furthermore, activation of antioxidant and detoxification systems allows tumor cells to tolerate high levels of ROS and escape cell death by systemic immune deregulation [91,92]. Indeed, tumor cells rely on ROS scavenging systems to induce pro-tumor eustress levels in the TME. In particular, it has been observed that inhibition of this system by glutathione can effectively shift the redox balance towards oxidative distress, which favors selective killing of tumor cells [93]. CRC progression occurs when the p53 gene is inactivated in combination with *APC* and *KRAS* mutations [85]. Changes in proliferation and nuclear accumulation of β-catenin are associated with activating *KRAS* mutations in the presence of additional inactivating mutations in *p53* and SMAD Family Member 4 (SMAD4), which promote the progression of adenoma to CRC. Loss of p53 increases tumor progression and is responsible for increased intestinal permeability associated with EMT and the induction of a pro-tumorigenic inflammatory microenvironment dependent on NF-kB [94]. Signaling driving tumor progression is involved in the control of defined metabolic pathways in CRC and other tumors [29] (Figure 2). The activity of Wnt signaling pathways is preserved by the metabolic gradient from glycolysis to mitochondrial oxidative phosphorylation in the crypt–villus axis. Physiological apoptosis dependent on APC requires the production of ROS by the mitochondrial respiratory chain [95]. Reduction or inactivation of APC and activation of Wnt/β-catenin signaling are early somatic events in CRC carcinogenesis. CRC progression is also associated with dysregulation of Wnt/β-catenin signaling and loss of individual APC functions. “Oxidative distress” represents a condition characterized by supra-physiological levels of oxidants or their inadequate detoxification/inactivation. Conditions of acute oxidative distress induced by H_2_O_2_, CRC cells and deprived of growth factors promote β-catenin expression and modulate the cytoplasmic APC protein [34]. Indeed, exposure to H_2_O_2_ induces differential gene expression, dependent on the cellular phenotype, to promote both Wnt/β-catenin-dependent and -independent signaling pathways. Primary CRC SW480 cells and corresponding metastatic SW620 cells respond differently to oxidative and metabolic distress by re-adapting the Wnt/β-catenin signaling pathway and promoting the mitochondrial isoform of APC that represses apoptosis. OS exposure decreases the levels of full-length APC and upregulates its shorter isoform [34]. Under conditions of mild/moderate OS (oxidative eustress), in which normal metabolism and steady-state functions require low levels of oxidants, primary and metastatic CRC cells differentially re-adapt Wnt/β-catenin signaling pathway, APC expression and their metabolic responses [35]. Oxidative eustress induced by H_2_O_2_ and JNK inhibition differentially regulates Wnt/β-catenin and APC expression in primary and metastatic CRC cells. Indeed, primary CRC cells SW480 are more responsive to H_2_O_2_ eustress combined with JNK inhibition since they show reduced viability compared to corresponding metastatic cells. Under eustress conditions, JNK inhibition reduces both glycolytic and respiratory capacity in metastatic SW620 cells, demonstrating a greater ability of the metastatic phenotype to adapt to TME [35]. Furthermore, oxidative eustress differentially modulates APC and β-catenin and mitochondrial oxygen consumption in primary and metastatic CRC cells, and JUN signaling may interfere with this response. These results show a differential modulation in the crosstalk between Wnt/β-catenin and JNK signaling pathways in primary and metastatic CRC cells under environmental eustress conditions. Moreover, metabolic reprogramming in CRC is associated with Wnt/β-catenin and APC mutation status and is closely linked to TME stress [35]. After APC loss, activation of Wnt determines the induction of TP53-induced glycolysis regulatory phosphatase (TIGAR) and Rac1. The former regenerates the levels of antioxidant GSH; the latter is an element of the NADPH oxidase complex implicated in ROS generation and is involved in the nuclear localization of β-catenin and the Wnt pathway [96,97] (Figure 2). Therefore, negative regulation of TIGAR and RAC1 may reduce intestinal cell proliferation. This indicates that Wnt activation may integrate two different ROS signals to support cell proliferation [97]. An intron-derived circMYH9 (hsa_circ_0092283) was found to be overexpressed in CRC cells and induce proliferation and serine metabolism. CircMYH9 increases endogenous serine production by modulating serine/glycine metabolism and redox homeostasis in a p53-dependent manner. CircMYH9 could promote cell proliferation in p53wt cells by degrading p53 pre-mRNA [98].

Deregulation of the Ras/Raf/MEK/MAPK/ERK pathway plays a pivotal role in CRC progression. Ras mutations constitutively activate the MAPK pathway, leading to aberrant cell proliferation and drug resistance. During tumor progression, oncogenic Ras supports pro-oxidant factors that induce activation of the response to DNA damage, dedifferentiation, genetic instability, hyper-proliferation, activation of subunits of the NADPH oxidase complex (NOX1/4), inactivation of antioxidants, ROS production from mitochondria or from cyclooxygenase-2 (COX2), resulting in additional mutations that stimulate tumor progression [87]. MAPK/JNK pathways are key regulators of the Warburg effect during tumor progression [99].

Endogenous and exogenous ROS activate phosphorylated protein/enzyme downstream signaling, including ERKs and JNK, in a mutual concentration-dependent manner by mixed lineage kinase 3 (MLK3, MAP3K11), a ubiquitously expressed mitogen-activated protein kinase kinase kinase (MAP3K) in the JNK pathway. Mathematical modeling revealed that MLK3 mediates a positive feedback loop (PFL), which equilibrates the ROS concentration-dependent signal flow between the ERK and JNK pathways (balancing point corresponding to the H_2_O_2_ concentration at which ERK and JNK phosphorylation is equivalent), and lead to cell proliferation or death [100]. In tumor cells exposed to low concentrations of ROS, ERK activity increases and proliferation is activated, while the MLK3-mediated PFL is not adequately stimulated to induce JNK. High concentrations of ROS induce JNK activation by promoting apoptosis and suppressing proliferation through ERK inhibition, while the MLK3-mediated PFL is activated [100]. Oxidative eustress and inhibition of JNK can differentially regulate the Wnt/β-catenin pathway and APC expression in primary and metastatic CRC [35]. JNK has been implicated in CRC progression, mainly through crosstalk between JNK and other signaling pathways, including the Wnt signaling pathway [101,102,103]. It is mostly activated by stress stimuli, which regulate different transcriptional activities and contribute to inflammation, apoptosis, cell proliferation, metastasis and angiogenesis in TME. Mitochondrial and cellular H_2_O_2_ is able to induce negative or positive regulation of aerobic glycolysis through phosphorylation of the JNK [35]. Upregulation of chemokine CXCL14 induced by H_2_O_2_ treatment promotes the CRC progression through the regulation of the EMT process and modulates the expression levels of cell cycle-related proteins (cyclin A1/B1, CDK1/2) and EMT-related proteins (E-cadherin, N-cadherin, vimentin) [104]. Moreover, the level of phosphorylated ERK (p-ERK) is higher in HCT116 cell lines expressing CXCL14 compared with HCT116/control cells, suggesting a role for this chemokine in the CRC cell proliferation and ROS-induced migration and in the treatment and prevention of CRC [104].

In the context of metabolic reprogramming of tumor cells, the altered signaling pathways influence cellular metabolism, particularly amino acid metabolism. To ensure the increased energy demand and facilitate the influx of essential amino acids required for their extremely rapid proliferation rate, different cancer cells selectively up-regulate specific amino acid transporters based on their molecular and metabolic profile [105,106]. Among transporters, SLC6A14 (ATB^0,+^) and SLC38A5 (SN2) cell-surface proteins provide substrates for cancer cell metabolic pathways and behave as signaling molecules. SLC6A14 is a Na^+^/Cl^−^-coupled transporter for 18 of the 20 amino acids that constitute proteins, including all the essential ones [106]. It intervenes in the entry of cystine into cells coupled to glutamate efflux out of cells and is involved in glutathione synthesis since it is the transporter of glycine, a constituent of glutathione. In this way, it protects tumor cells against oxidative stress [105]. Indeed, SLC6A14 is expressed at basal levels in the normal colon but is up-regulated in colon cancer [107]. The functional activity of SLC6A14 is also coupled to mTOR activation since leucine is a strong activator of the oncogenic mTOR signaling pathway [106]. Moreover, SLC6A14 is a transcriptional target for TCF4/β-catenin and is up-regulated by the canonical Wnt signaling pathway in CRC [105]. On the other hand, SLC38A5 is a Na^+^-coupled transporter with very restricted specificity towards some amino acids: it mediates the uptake of only asparagine, histidine, glutamine, serine, glycine and methionine [106]. In particular, SLC38A5 acts as a Na^+^/H^+^ exchanger since it is coupled to H^+^ efflux from cells. Thus, SLC38A5 prevents intracellular acidification resulting from the excessive generation of lactic acid in cancer cells by mediating the transfer of amino acids and Na^+^ into cells, leading to the removal of intracellular H^+^. This effect results in intracellular alkalinization, which promotes macropinocytosis, a non-selective mode of uptake nutrients, such as proteins, from the extracellular environment, representing another mechanism for the import of amino acids in cancer cells in addition to amino acid transporters that are upregulated in cancer [106,108]. SLC38A5 is expressed in the intestinal tract, but little is known about its involvement in colon carcinogenesis [108]. In a CRC cell model, the oncogenic mutation G12D in KRAS increases SLC38A5 expression by Myc and represents the driver of macropinocytosis [108]. KRAS G12D mutation also induces the expression of the amino acid transporter SLC7A11 that protects cancer cells from ferroptosis (an anti-growth process) since this genetic alteration increases cellular levels of glutathione (Figure 2). The expression of this transporter is suppressed by loss of p53 in CRC [108]. Reprogramming of cysteine metabolism in colorectal carcinogenesis is induced by hypoxia. Elevated ROS levels in the tumor microenvironment increase exogenous cystine/cysteine uptake by activating transcription factor 4 (ATF4) to maintain elevated intracellular cysteine levels in CRC and support tumor growth. Furthermore, hypoxia-induced ROS generation upregulates cystine and cysteine transporters (SLC7A11, the light chain of cystine/glutamate antiporter system; SLC3A2, the heavy chain of cystine/glutamate antiporter system; SLC1A4, alanine/serine/cysteine/threonine transporter 1; SLC1A5, alanine/serine/cysteine/threonine transporter 2) via ATF4. Overexpression of glutathione synthase (GSS) rapidly increases the flux from cysteine to reduced GSH to support tumor development by scavenging excessive ROS and maintaining them at lower cytotoxic levels to promote CRC progression [109]. SLC25A39, a mitochondrial membrane carrier, plays an important role against OS and in regulation of the mitochondrial redox state by transporting GSH into mitochondria from the cytoplasm [110]. In CRC, overexpression of SLC25A39 is not only correlated with decreased ROS concentration, but it also induces cell proliferation and migration and inhibits apoptosis. Increased levels of SLC25A39 are also associated with low expression of immune checkpoints and reduced response to immunotherapy. Conversely, SLC25A39 knockdown is correlated with inhibition of CRC cell survival and migration in addition to reduced mitochondrial GSH import and ROS increase in levels, resulting in the inability of cells to eliminate or neutralize produced ROS in the mitochondria [110] (Table 2).

In the TME, ROS produced by activated T and NK cells recruit neutrophils and macrophages to kill tumor cells [38]. Myeloid-derived suppressor cells (MDSCs) are involved in tumor progression since they suppress CD8+ T cell proliferation by nitration of T-cell receptors (TCRs) (ROS-dependent generation of peroxynitrite) [111]. CRC recurrence/relapse has been ascribed to altered ROS levels generated in the microenvironment of cancer cells [112]. Based on ROS concentration, OS increases cancer progression, leading to therapy resistance or inducing cancer cell death. In progression and metastasis steps, cancer cells adapt to high ROS levels by increasing NADPH production [27,49]. Different proteins are involved in cellular redox homeostasis. Their deregulation is linked to CRC progression. USP11, a deubiquitinating enzyme associated with the proteasome, regulates the balance in the production and elimination of ROS and stabilizes the transcription factor NRF2, protecting it from degradation [113]. USP11 is overexpressed in CRC, where it acts as an oncogene. It inhibits mitochondrial apoptosis and activates CRC progression through the binding of NRF2 to the antioxidant reaction element (ARE) in the USP11 promoter to activate its transcription [113]. TAMs induce or inhibit tumor immunity through polarization into M1 and M2 types. During carcinogenesis, TAMs initially show M1-like polarization to limit tumor growth through an increased elimination of cancer cells as well as NF-κB signaling pathway activation and generation of pro-inflammatory cytokines, ROS and reactive nitrogen species (RNS). During cancer progression, TAMs move toward an M2-like polarization state, which promotes tumor growth through the production of different pro-tumorigenic and immunosuppressive cytokines and anti-inflammatory factors. ROS can activate both M1 and M2 populations. Tumor-associated neutrophils (TANs) and their N1 and N2 phenotypes are also involved in cell proliferation, invasion and metastasis [114]. In tumors that arise from chronic inflammation, such as CRC, ROS produced by TAMs may show a different impact on progression compared to tumors not associated with inflammation [38].

**Table 2:** Target molecules or pathways activated by ROS, effects on cells and cellular responses in CRC progression.

CRC Progression			
Target Molecules or Pathways Activated by ROS	Effect	Cell Response	References
Wnt/β-catenin	induction of TIGAR and RAC1	TIGAR regenerates the antioxidant GSH levels; RAC1 involved in ROS generation and nuclear localization of β-catenin	[96,98]
circMYH9	overexpression of circMYH9; increased serine production and redox homeostasis	increased cell proliferation	[98]
KRAS mutations	activation of MAPK pathway; ROS production from mitochondria or COX2 resulting in additional mutations	drug resistance; activation of response to DNA damage, de-differentiation, genetic instability, hyperproliferation, activation of NADPH oxidase complex, inactivation of antioxidants	[87]
KRAS, p53 and SMAD4 mutations	changes in cell proliferation and nuclear accumulation of β-catenin	progression of adenoma to colon cancer	[94]
ERK and JNK in a concentration-dependent manner by MLK3	equilibrium in the signal flow dependent from ROS concentration between the ERK and JNK pathways	increased cell proliferation	[100]
Loss of p53	induction of protumorigenic inflammatory microenvironment NF-kB-dependent	increased gut permeability and EMT	[94]
JNK	regulation of transcriptional activities; interplay with Wnt signaling and other pathways	inflammation, apoptosis, cell proliferation, metastasis, and angiogenesis in TME	[35,102,103]
MAPK/JNK pathways	regulation of the Warburg effect	tumor progression	[102]
CXCL14 upregulation	regulation of EMT process and expression of cyclin A1/B1, CDK1/2 and E-cadherin, N-cadherin, vimentin	CRC progression	[104]
Increased exogenous cystine/cysteine uptake by SLC7A11, SLC3A2, SLC1A4, SLC1A5, SLC25A39	process mediated by ATF4	high intracellular cysteine levels; lower cytotoxic levels of ROS to support CRC progression	[109]
USP11 deubiquitinating enzyme overexpression	USP11 role in CRC as an oncogene	inhibition of mitochondrial apoptosis	[113]

## 4. ROS Detection and Identification

OS is measured by malondialdehyde (MDA), reduced GSH, myeloperoxidase (MPO), sulfhydryl (SH-) and SOD activity [115].

MDA is an end-product of lipid peroxidation of membrane PUFAs induced by ROS. Although there are numerous techniques for measuring MDA, not all demonstrate specificity and accuracy. MDA is quantified spectrophotometrically using thiobarbituric acid reactive (TBARS) assay, but this test is not specific for MDA since other aldehydes can form light-absorbing products in the same range as MDA [116]. Enzyme-linked immunosorbent assay (ELISA) shows increased specificity compared to TBARS, while the high-pressure liquid chromatography (HPLC) method for MDA measurements is more accurate and sensitive than the spectrophotometric TBARS. Reverse-phase high-performance liquid chromatography using 2,4-dinitrophenylhydrazine as a derivative reagent is more accurate for the quantification of MDA compared to the modified 2-thiobarbituric acid (TBA) spectrophotometric method [117]. Measurement of GSH and related intermediates is crucial in the evaluation of the cell redox and metabolic status in vivo and in vitro. Many systems measure the level of GSH, such as HPLC, bioluminescence, fluorometric methods, liquid chromatography–mass spectrometry and gas chromatography–mass spectrometry. Another spectrophotometric method is based on the oxidation of GSH in oxidized glutathione (GSSG) by the sulfhydryl reagent 5,5′-dithio-bis(2-nitrobenzoic acid) (DTNB) [118]. MPO is a heme enzyme present in granules of inflammatory cells. MPO function can be measured spectrophotometrically by peroxidase activity assays [119]. MPO in tissue samples is measured by different techniques, including ELISA or quantitative real-time polymerase chain reaction (qPCR) for mRNA expression, while kinetic assays are used to evaluate the enzymatic activity. In a kinetic colorimetric assay, MPO is determined in intestinal tissue supernatant [120].

Sulfhydryl (SH-) is contained in proteins. Molecules with SH- groups are described as thiols or mercaptans. Proteins containing Cys residues are components of enzymes, organelles, intracellular and extracellular membranes [121]. The formation of a disulfide bridge between a reactive cysteine residue and the abundant cellular tripeptide glutathione is a stable yet reversible reaction, defined as S-Glutathionylation [119]. A colorimetric assay by a microplate reader is used for the determination of sulfhydryl group/total thiol (-SH). Finally, SODs are metalloenzymes that catalyze the dismutation of superoxide anion into oxygen and hydrogen peroxide. SOD assay can be carried out by a method using nitro blue tetrazolium. This is the substrate that reacts with superoxide anions produced in the presence of methionine as an electron donor to produce formazan, which is a blue-colored complex [122]. However, different methods have been developed to monitor extracellular and intracellular ROS production. Plasma concentrations of ROS are difficult to measure [123]. ROS production might be analyzed after blood centrifugation and serum separation. Fluorescence and electrochemical methods are sensitive and selective for detecting ROS in live cells and are different depending on the type of ROS [124]. Indeed, one of the most used methodological approaches is based on the detection of ROS-sensitive fluorescent probes in living cells (real-time detection) by a fluorescence microplate reader [125]. Dihydroethidium (DHE), MitoSOX Red and 5-(and 6)-chloromethyl-2′,7′-dichlorohydrofluorescein diacetate (CM-H2DCFDA) are the fluorescence probes used in adherent cells to detect cytosolic superoxide anion O2•–, mitochondrial O2•– and H_2_O_2_ production, respectively [125]. At the cellular level, •OH and H_2_O_2_ can be measured using the fluorogenic probe 2′,7′-dichlorodihydrofluorescein diacetate (H_2_DCFDA). It crosses the cell membrane and is hydrolyzed to form 2′,7′-dichlorodihydrofluorescein (H_2_DCF), which reacts with intracellular H_2_O_2_ to generate 2′,7′-dichlorofluorescein (DCF), which can be analyzed by flow cytometry or by a fluorescence plate reader at λ = 530 nm [126,127]. Detection of O2•– is difficult since it is highly reactive and exhibits a short half-life. Therefore, real-time detection techniques are crucial to detect O2•– at elevated concentrations. Fluorescence and electrochemical methods are able to measure O2•– at the generation source since they penetrate through cells, mitochondria or cellular compartments [124]. Detection of O2•– by fluorescence is ambiguous because both DHE and MitoSOX produce ethidium, a non-specific oxidation product, and the O2•−-specific product 2-hydroxyethidium, that show overlapping fluorescence spectra [127]. Limitations for in vivo detection include the biological damage consequent to high-energy light emission. On the other hand, electrochemical techniques are based on the same principle as the fluorescence method, as they associate electrochemical reactions with sensing elements to identify O2•–. Biological catalysts (enzymes), such as SOD and cytochrome-c (Cyt-c), are commonly used in electrochemical methods and allow fast dismutation of O2•– [124]. Compared to O2•–, H_2_O_2_ has a longer lifetime, up to minutes, crosses cell membranes and distributes into cellular compartments. In the electrochemical techniques for H_2_O_2_ detection, the electrode surface is modified with an electrocatalyst represented by an inorganic or an organic compound, such as Cyt-c, horseradish peroxidase, hemin or myoglobin. In live cells, bimetallic electrochemical sensors are more often used for the detection of H_2_O_2_ because of their higher catalytic activity [124]. High sensitivity and specificity for H_2_O_2_ are obtained by incorporation of appropriate targeting gene sequences, from which originate genetically encoded fluorescent protein sensors containing a dithiol switch that modifies the overall fluorescence of the probe based on oxidation status. These probes can be directed to different cell compartments and have been realized by coupling a redox-sensitive green fluorescent protein mutant to an H_2_O_2_-sensitive thiol protein, such as oxyR [127]. For •OH detection, usual techniques comprise electron paramagnetic resonance spectroscopy, chemiluminescence, fluorescence and electrochemical methods. •OH shows high reactivity and a short lifetime of about 9−10 s. The fluorescence method detects •OH in living cells through real-time analysis [124]. Conversely, amplex red (10-acetyl-3,7-dihydroxyphenoxazine) is a nonfluorescent derivative of dihydroresorufin for detecting ROS in experimental systems. Amplex red is cell impermeable and is used to measure ROS in isolated organelles, such as mitochondria, or to investigate the amount of extracellular H_2_O_2_ released [128]. Fluorescence microscopy, from wide-field to spinning disk confocal microscopy, is used to detect ROS in non-homogeneous samples, such as differentiating cells or manipulation of gene expression, or for the analysis of non-homogeneous subcellular compartments. Common dyes used for measuring ROS production for confocal and wide-field microscopy are DHE, MitoSOX Red and CM-H2DCFDA, which are oxidized by ROS inside cells. The kinetics of their accumulation exactly represent the rate of ROS production [125].

## 5. Implications of ROS Production for CRC Treatment

Following a better understanding of the molecular interactions of ROS in CRC development and progression, much attention has been given to the development of strategies for the therapeutic modulation of ROS in the treatment of CRC. However, ROS production may promote CRC progression and/or recurrence through dysregulated inflammatory immune mechanisms. A comprehensive review on ROS-induced cancer cell death in the treatment of CRC was conducted by Yang Zhang and colleagues [129]

Damage and cell death can result from excess ROS [130]; in CRC, this effect is a major mediator of chemotherapy- and radiotherapy-induced DNA damage [131,132]. Although CSCs are less sensitive to ROS-generating therapies, they are thought to be responsible for cancer recurrence [133]. These topics have been covered in previous reviews, and researchers are encouraged to read [129].

Extreme OS from very high levels of ROS induce greater vulnerability of cancer cells to external agents and can play an anti-tumor role, resulting in the impairment of cell components, cell-cycle arrest and apoptosis or autophagy as a consequence of mitochondrial and DNA damage through activation of p53 [25,49,134,135,136]. Added to these, there is an altered crosstalk between infiltrating immune cells, increased peroxisomal activity, hyperactivation of enzymes, including oxidases, cyclooxygenases and lipoxigenases and alterations in redox balance affecting the removal of ROS by the intracellular antioxidant protective system [24,137]. In CRC cells, CD40L (CD154), the ligand of CD40 and a member of the tumor necrosis factor receptor (TNFR) superfamily, triggers an ROS-dependent apoptotic pathway by the activation of tumor necrosis factor receptor-associated factor 3 (TRAF3)/NOX/apoptosis signal-regulating kinase 1 (ASK1) and p38/JNK signaling to stimulate the caspase-9-dependent mitochondrial pathway or to induce upregulation of intracellular TRAIL in caspase-10-associated apoptosis [138]. ROS influence the adhesion of tumor cells to mesothelial monolayers. After preincubation of the mesothelium with PMNs, the adhesion of colon carcinoma cells increased together with an up-regulation of intercellular adhesion molecule 1 (ICAM-1), CD44, and vascular cell adhesion molecule 1 (VCAM-1) in a model using Caco-2 colon carcinoma cells [139]. Specifically, ROS can damage the mesothelial layer by inducing cellular membrane rupture, DNA damage or adenine nucleotide depletion. ROS up-regulate the cell surface adhesion proteins and increase tumor cell adhesion in the liver with mechanisms that can be inhibited by treatment with Edavarone [139,140]. In addition, increased ROS levels show a transient destructive effect on electrical impedance and the formation of intracellular gaps, facilitating tumor cell adhesion [140]. The development of CRC metastasis also depends on the higher levels of ROS that are produced during surgical trauma. After major surgery such as colectomy, nephrectomy, prostatectomy and adenectomy, ROS increase significantly at 24 h and 72 h postoperatively [123]. Surgical resection is the gold standard of therapy for CRC, but surgical trauma itself contributes to CRC recurrence and metastasis in 25–50% of patients resected of the primary CRC [140].

### 5.1. Surgery

Any type of surgical trauma results in higher levels of OS [115,141,142]. Open abdominal surgery, considered a more systemic stressful trauma, increases OS in contrast to laparoscopic procedures that are associated with a reduced immune response. ROS represent a valid indicator of the severity of surgical treatment [123]. Interestingly, patients treated by laparoscopy-assisted colectomy show a higher probability of cancer-related survival than those treated by open surgery when long-term results of laparoscopy-assisted colon resection were compared with the open colon resection for nonmetastatic CRC [143]. ROS production is higher in patients who underwent intermediate-risk surgery than in the low-risk surgery group [123]. The severity of surgical trauma correlated with survival outcome with not completely defined mechanisms induced by ROS activation. Pneumoperitoneum is considered an important factor in preserving intra-operative and post-operative immune response during laparoscopic surgery. Carbon dioxide insufflation, used during laparoscopic surgery, reduces PMN-stimulated ROS production [144]. ROS levels are reduced in blood samples of patients who underwent laparoscopy after CO_2_ incubation. These effects were evident after 15 min and reached statistical significance after 2 h exposure. Although the mechanisms involved in the immunomodulation by CO_2_ are not fully understood, in vivo results have demonstrated the crucial effects of CO_2_ pneumoperitoneum on the function of PMN in concomitance with hypoxia and acidification [144]. Higher ROS levels are associated with abdominal pain after surgical resection. A linear correlation is observed between ROS production and morphine consumption, as measured by fluorescence in patients who underwent intermediate-risk surgery [123]. Surgical stress is related to OS [145]. ROS negatively affects tissue repair mechanisms. In CRC surgery, colorectal anastomotic dehiscence represents a severe complication in common clinical practice. Increased levels of ROS correlate with colorectal anastomotic dehiscence [145]. After the analysis of human colon and rectum tissues, it was observed that rectal tissue was more damaged than colon tissue, as documented by the increased levels of ROS [145]. An OS index-based score for prognosis in CRC patients treated surgically was recently proposed and validated [142].

Based on biochemical indicators of OS indices such as albumin, total bilirubin, direct bilirubin, uric acid and blood urea nitrogen levels, a relationship with prognosis of CRC-treated patients was established. Indeed, these biomarkers derived from systemic oxidative stress in peripheral blood may have an impact on the prognosis of CRC. Low levels of total bilirubin, blood urea nitrogen and elevated levels of uric acid have a positive effect on OS, while low levels of uric acid are associated with a worse prognosis in CRC patients. Bilirubin, uric acid and blood urea nitrogen are indicators of liver and kidney function. Bilirubin has antioxidant properties and is considered an anticancer factor. High levels of total bilirubin are associated with poor prognosis in CRC. Hyperuricemia associated with OS leads to DNA damage and is involved in the production of oxidative and inflammatory factors and apoptosis [146]. Renal oxidative stress responses can also be measured using serum and urine biomarkers. One of the oxidative stress indices is 8-OH-dG, which increases in urine after renal oxidative stress [147]. The CRC-integrated OS score (CIOSS) is a predictive model of outcome. It is a CRC-specific prognostic indicator derived from a combination of oxidative stress markers. CIOSS analyzes the relationship between oxidative stress indices such as albumin, total bilirubin, direct bilirubin, uric acid, blood urea nitrogen and prognosis in patients with CRC [142,146]. Patients with high CIOSS had worse overall survival and worse disease-free survival compared with patients with a low value [142].

### 5.2. Prognosis

OS predicts CRC prognosis based on tumor stage and has an important influence on premature death in CRC patients. In a population of 123 patients who underwent CRC surgical resection, a reduction in reactive oxygen metabolite derivatives (d-ROMs) was reported following surgical resection. In addition, correlations were documented among d-ROMs and CRC tumor size, depth of invasion, lymph node metastasis and advanced stage (II and III CRC stage). In other words, a large CRC produces more ROS and increases systemic OS levels [141]. As supported by the results of in vitro studies, hemoglobin may increase CRC cell proliferation by release of ROS [148]. In addition, the administration of hemoglobin negatively impacts the cytotoxic effects of 5-fluorouracil and 5-deoxy-5-fluorouridine [148].

### 5.3. ROS as a Therapeutic Strategy in CRC

Recent research has also focused on H_2_O_2_-related diagnostics based on nanomaterials and technologies that use ROS production or H_2_O_2_ consumption as sensing modalities [149]. In breast cancer research, novel molecular theranostics have been synthesized as targeted cancer therapeutics and imaging agents [150]. These molecules are effective in killing tumor cells with high levels of H_2_O_2_ while being safe for normal epithelial cells. This new frontier of biomedical science is theranostic nanomedicine. The concentration of H_2_O_2_ can alter the fluorescence signal during fluorescence imaging of tumors. Interestingly, the H_2_O_2_-induced CO bubbles could be used as a contrast agent for ultrasound imaging. Indeed, to enhance the ultrasound signal, polycarbonate nanoparticles can generate CO2 nanobubbles by oxidizing esters with H_2_O_2_ [149]. Theranostic nanomedicine applications are a revolutionary approach to improving CRC diagnosis and therapy [151]. For example, the nanotechnology-based formulation, thiolated chitosan and 5-FU nanoencapsulation is non-toxic and has improved chemotherapeutic efficacy in CRC patients [151]. In addition, improving the efficacy of therapies using biomimetic nanocarriers by targeting the TME rather than tumor cells alone could effectively improve patient’s therapeutic response and maintain biosafety. Nanodrug platforms with redox properties can have multi-enzyme activity with a cytotoxic role in tumor tissue by catalytically oxidizing their substrates to produce harmful reactive oxygen species such as hydroxyl radicals [152]. Indeed, loaded nanoparticles (NPs) resulted in increased ROS production and GSH depletion, induced ferroptosis and suppressed glycolysis in CRC cells. In vivo, the NPs significantly inhibited tumor growth, with a synergistic effect of photothermal and chemodynamic therapy [153,154]. Oral nanomaterials show promising potential in modulating intestinal immune cells, reducing inflammation of the gut and modifying microbiota in the therapeutic approach of primary CRC. A multifunctional oral dextran–aspirin nanomedicine (P3C-Asp) was constructed with aspirin, an ROS scavenging fraction and prebiotic agent for the treatment of primary CRC [155]. It releases salicylic acid (SA) in response to high ROS levels, scavenging ROS to reduce inflammation with SA and modulate gut microbiota with the prebiotic dextran. Oral P3C-Asp reduces cancer-associated inflammation by increasing SA accumulation in CRC tissues and scavenging ROS. Moreover, P3C-Asp stimulates microbiota homeostasis, with a significant reduction in pathogens [155]. Most therapeutic evaluations of nanocarriers for CRC have been conducted in animal studies, so further preclinical validation studies in humans are needed.

Unfortunately, resistance to chemotherapy, radiation therapy or targeted therapy represents a significant barrier in adjuvant treatments of CRC. Comprehension of the mechanisms responsible for resistance permits the development of new therapeutic strategies with the aim of overcoming it [156]. Promising photodynamic nanoplatforms could prevent chemotherapy resistance in CRC through the manipulation of osmotic pressure and redox homeostasis [129].

A new therapeutic approach is represented by photodynamic therapy (PDT), a noninvasive modality to locate and destroy CRC cells by producing ROS and inducing oxidative stress. In this method, which is known as dark toxicity, the photosensitizers that are located in the tumor generate highly cytotoxic reactive oxygen species until the tumor cells die [157]. PDT mediated by hypericin (HY-PDT), a photosensitizer with photochemical properties, might potentiate the cytotoxicity of oxaliplatin (L-OHP) in CRC cells by an ROS-dependent mechanism involving drug efflux, GSH-related detoxification and nucleotide excision repair (NER)-mediated DNA repair [158]. Since the dark toxicity of photosensitizers and inadequate penetration of light limit clinical applications of PDT, a new photosensitizer 5-(4-amino-phenyl)-10,15,20-triphenylporphyrin with diethylene-triaminopentaacetic acid (ATPP-DTPA)-mediated PDT (ATPP-PDT) was evaluated at the irradiation of a 450 nm blue laser in CRC treatment. This novel method inhibits tumor growth and induces apoptosis by the involvement of the p38 MAPK pathway in CRC cells in vitro as the effect of the generation of elevated levels of ROS [159]. Furthermore, traditional PDT has been improved through X-ray-induced photodynamic therapy (X-PDT), in which X-ray imaging technology has evolved after the introduction of X-ray luminescence nano-agents, which increase detection sensitivity due to their almost unlimited depth in living tissues [160]. The use of ROS as a new possible therapeutic strategy is based on the manipulation of the cellular redox balance through the relationships among ROS, FAD/NADH, NADH and NADPH and caspase-3 activity, using multiparametric time-lapse microscopy [161]. Two-photon excitation fluorescence lifetime imaging microscopy (FLIM) was used to monitor apoptosis through the genetically encoded FRET-based sensor of caspase-3, mKate2-DEVD-iRFP and the autofluorescence of redox cofactors in CRC cells after induction of apoptosis with staurosporine, cisplatin or hydrogen peroxide. All three agents activate apoptosis through the mitochondrial pathway. Regardless of the apoptotic stimulus used, increases in ROS are correlated with enzyme-bound NADH and caspase-3 activation [161]. Sodium butyrate (NaB), a histone acetylation inhibitor produced by intestinal flora, increases ROS levels by reducing GSH in cancer cells. Moreover, it shows inhibitory properties on the proliferation of CRC cells, induces apoptosis in vitro and delays tumor progression in vivo by the activation of the mitophagy pathway. The combined treatment of NaB and 5-fluorouracil (5-FU) shows better therapeutic results than monotherapy and increases different bacterial species in the gut microbiota, improving the outcomes and prognosis of CRC patients and reducing the adverse effects of 5-FU [162].

Hypoxia-inducible factor 1α (HIF-1α) induces glycolysis and the PPP in CRC cells as well as 5-FU resistance, which is driven by ROS-induced PI3K/Akt and Wnt/β-catenin signaling pathways. The Wnt/β-catenin pathway positively regulates HIF-1α, increasing the expression of downstream glycolysis and PPP-related genes. HIF-1α is a potential biomarker for 5-FU-resistant CRC. Targeting HIF-1α in combination with 5-FU may represent an efficient therapeutic strategy in 5-FU-resistant CRC [163].

Gypenosides (Gyp), the major components isolated from Gynostemma pentaphyllum, induce apoptosis in CRC cells through the mitochondria-dependent pathway and show chemo-sensitization in increasing the anti-tumor effect of 5-Fu in vitro and in vivo, with little side effects [164]. Indeed, Gyp or 5-Fu + Gyp treatment significantly increases intracellular ROS levels and DNA damage [164].

Metformin (MET), the first-line therapy for type 2 diabetes mellitus, reverses chemoresistance in CRC cells when combined with cisplatin (CDDP). It increases the chemosensitivity of SW480 and SW620 cell lines to CDDP, inhibits cell proliferation and induces apoptosis through the PI3K/AKT signaling pathway mediated by ROS [165]. Moreover, MET sensitizes HCT116 and SW480 cell lines to cytotoxicity induced by irinotecan (CPT-11) and arrests the cell cycle in the G1 and S phases [166].

Long noncoding RNAs (LncRNAs) AP002387.2 (lnc-AP) could sensitize the HCT116/Oxaliplatin (L-OHP) and SW480/L-OHP cell lines to L-OHP by encoding short peptide pep-AP, which sensitizes CRC cells to L-OHP in vitro and in vivo [167]. The pep-AP/transaldolase 1 (TALDO1) pathway suppresses PPP, reducing NADPH/NADP+ and GSH levels and increasing ROS levels and apoptosis by inhibition of the enzyme of PPP TALDO1 [167]. Salvianolic acid B (SalB), isolated from Salvia miltiorrhiza Bge, reverses multidrug resistance (MDR) in HCT-8/VCR cells by increasing intracellular ROS levels, which induce apoptosis and downregulate the expression of P-glycoprotein (P-gp) [168]. BaP1, a phenoxazine derivative, reduces cell proliferation, survival and cell migration through ROS generation, apoptosis promotion and angiogenesis inhibition. It represents a promising candidate to overcome resistance to chemotherapy and radiotherapy in CRC [169].

Radiotherapy, especially X-rays, exerts its cytotoxic effects on tumor cells, mainly through the generation of ROS. Tumor cells counteract the damage induced by ionizing radiation by increasing DNA repair and anti-oxidation defense, controlling ROS-induced damage. Promising therapeutic approaches include the inhibition of anti-oxidant pathways or the production of high oxidative stress, combining ROS-generating agents and radiotherapy, such as erastin, which is able to increase the sensitivity of chemotherapy and radiotherapy [170,171]. Piperlongumine (PL), a natural alkaloid extracted from the fruits of the plant species *Piper longum* L., modifies both hypoxic and intrinsic radioresistance of CRC cells, leading to enhanced radioresponse, slowing tumor growth and improving the survival rate of tumor-bearing mice [172]. PL selectively kills tumor cells through the alteration of ROS homeostasis due to the increased production of ROS as a result of the inhibition of antioxidant systems, including the decrease in GSH and the inhibition of TrxR. The cytotoxic effect of PL is associated with augmented ROS-induced DNA damage, cell cycle arrest in the G2/M phase, induction of apoptosis and autophagy and inhibition of cellular respiration [172].

Dihydroartemisinin (DHA), a semi-synthetic derivative of the natural compound artemisinin and a first-line antimalarial drug, is able to activate the protective Keap1/Nrf2 pathway in HCT116 CRC cells [173]. This signaling pathway controls the intracellular availability of cysteine through upregulation of SLC7A11, as well as the synthesis of glutathione. In a therapy combined with ionizing radiation, DHA improves the protective antioxidant defense in CRC cells. To extend the combined effects of DHA and radiotherapy, additional use of an inhibitor of the Keap1/Nrf2 pathway or of signaling pathways controlling intracellular glutathione should be contemplated [173].

The efficacy of therapies in CRC is influenced by the complex network of signaling pathways involved in carcinogenesis, which influence the malignant phenotype, the immune response and the TME [174]. Since MAPK/ERK, PI3K/Akt and JNK pathways, as well as the Wnt/β-catenin and RAS-ERK signaling, are interconnected, it is possible to develop effective multi-targeted therapeutic approaches in the treatment of CRC [14,127]. Moreover, modulation of the response to oxidative stress in deregulated signaling pathways could promote new personalized therapeutic strategies in CRC to adapt therapies to the individual molecular profile of a tumor. Targeted therapies represent a precision medicine approach and are planned to interfere with specific molecular targets that are involved in tumor growth, progression and metastasis [175]. They reduce the damage to normal cells, offering better efficacy and decreasing symptoms of chemo toxicity. Among monoclonal antibodies (mAbs) for CRC, Cetuximab and Osimertinib target EGFR, Trastuzumab and Neratinib are designed for HER2/HER3 positive cancers, AMG510 is an inhibitor for KRAS, tyrosine kinase inhibitors Binimetinib for MEK1, and Alpelisib and Copanlisib target the PI3K pathway, although their effectiveness is often hampered by intrinsic or acquired resistance mechanisms. Intrinsic resistance might originate from mutations, loss of heterozygosity or gene amplification, while extrinsic resistance includes acquired mutations in the EGFR genomic alterations in RAS, BRAF, HER2 and MET [174]. Patients with RAS wildtype/BRAF mutations show activation of the RAS/RAF/ERK signaling pathway and resistance to anti-EGFR monoclonal antibodies [176]. The biomarker profile involving KRAS/NRAS/BRAF/PIK3CA mutations, as well as PTEN loss and amplification of HER2, are predictive of resistance to anti-EGFR therapy [174]. Recently, the therapeutic effect of small-molecule Wnt-inhibitors, such as ICG-001 on cancer stemness and metastasis, was evaluated [177]. Metformin and ICG-001 act synergistically and overcome cancer stem-like cell resistance mediated by 5FU in CRC by promoting autophagy and apoptosis [178]. Furthermore, mechanisms of drug resistance, such as upregulation of alternative Wnt ligands and receptors or mutations in molecular components of the pathway, must be better understood [174].

Unlike chemotherapy, radiotherapy and surgery, immunotherapy is able to manage the initiation, growth and progression of cancer by activating the patient’s immune system to kill tumor cells. Clinical trials to evaluate the efficacy of combined targeted therapies with immune checkpoint inhibitors, such as antibodies blocking the cytotoxic T lymphocyte-associated protein 4 (CTLA-4) or the programmed cell death 1 (PD-1) pathway, alone or in combination are ongoing [174,179]. CRC patients with microsatellite instability-high (MSI-H) and mismatch repair deficiency (dMMR) show a positive response to PD1/programmed death-ligand 1 (PDL1) blockade treatment [180]. Nevertheless, only in a small percentage of patients affected with advanced/metastatic CRC, PD-1/PDL1 inhibitor therapy significantly improves overall survival [181]. A study evaluated the effects of Anlotinib (anlo) on TME in CRC and its effects in combination with immune checkpoint inhibitor therapy [182]. Anlo is a multitarget tyrosine kinase inhibitor (TKI), which simultaneously inhibits vascular endothelial growth factor receptor (VEGFR) 2/3, fibroblast growth factor receptor (FGFR) 1-4, and platelet-derived growth factor receptors (PDGF-R) α/β. The PD1/PDL1 pathway mediates immunosuppression and tumor immune escape; thus, when PD1-mediated inhibitory signals are activated by its ligand PDL1, the functions and proteins of immune cells decrease [183]. Cytotoxic effects of anlo alter intracellular redox homeostasis, induce intracellular oxidative stress and enhance ROS levels to activate the JNK/AP-1 pathway, which upregulates the expression of PDL1, interferon (IFN)-α/β/γ and the C-X-C motif chemokine ligand 2 (CXCL2). These molecules, in turn, might contribute to the upregulation of NK cells and M1 macrophages. Moreover, a synergistic therapeutic effect has been demonstrated when anlo is combined with immune checkpoint inhibitor PDL1 [182]. Personalized medicine, especially the personalization of ROS-mediated treatments, could significantly improve cancer therapy by understanding the re-dox-associated genes in CRC and predicting specific treatments. Genomic, proteomic, and metabolomic analyses could identify specific molecular alterations that could be used for targeted therapies and personalized treatments to stop cancer progression and prevent relapse/recurrence after treatment. Tumor cells may be well adapted to OS and exhibit antioxidant capacity, which may have therapeutic implications. Combining radiotherapy or chemotherapy with drugs that deplete antioxidant systems in tumor cells may be a novel therapeutic option. Strategies that modulate OS may be an applicable approach to reducing chemoresistant tumor cells. The effects of ROS manipulation on different stages of CRC are not yet known, nor is the efficacy of ROS-based therapies on different patient genotypes. Identification of redox phenotypes in patient biopsies may provide an opportunity to modulate redox-related pathways and targets with therapy and to assess treatment sensitivity. The use of anti-ROS treatments raises questions regarding the selectivity and safety of drugs used in the therapeutic approach. The effectiveness of gene targeting strategies is, unfortunately, limited by drug resistance and genomic instability acquired by tumor cells.

## 6. Conclusions

ROS physiologically regulates intestinal epithelial cell proliferation and differentiation in a complex network of interactions modulated by the functional demands of the tissue microenvironment, triggered by external and internal stimuli to the intestinal barrier itself. These interactions in the TME can be appropriately re-modulated by ROS to promote tumor initiation, progression, invasion, dissemination and metastasis. Redox-dependent dysregulation of protein function favors the accumulation of genetic mutations that are functional for the new metabolic requirements of tumor cell growth and survival. OS influences treatment resistance in CRC, and a better understanding of how this occurs will allow the development of new personalized therapeutic approaches to overcome resistance. Understanding the molecular dynamics underlying the involvement of ROS in CRC treatment would also allow an integrated approach of different precision theranostic options to reduce damage to healthy tissue for improved efficacy and reduced toxicity. Integrated clinical trials will be needed to effectively translate selective multi-target therapeutic approaches adapted to the molecular profile of the tumor and the immune status of the patient into clinical practice.

## Figures and Tables

**Table 1:** Oxidative molecules and target molecules or pathways activated by ROS; effects on cell and cellular responses in CRC initiation.

CRC Initiation			
Oxidative Molecules	Genotoxic Effects of ROS	Cell Response	References
8-oxodG	oxidative DNA damage	activation of OGG1 and MUTYH	[55]
**Target molecules or pathways activated by ROS**	**Effect**	**Cell response**	**References**
Wnt/β-catenin	deficit of β-catenin degradation	activation of carcinogenesis target genes	[76]
KRAS mutation	activation of Raf-MEK-ERK and PI3K-AKT pathways	superoxide production by upregulation of NOX1	[85]
		resistance to apoptosis	[86]
		changes in intracellular metabolism; activation of pro-oxidant pathways resulting in additional mutations	[87]
EGFR	activation of PI3K pathway	proliferation, survival, migration, invasion, angiogenesis, et al.	[76]
PTEN cys124	activation of PI3K/AKT pathway Wnt/β-catenin pathway deregulation	cell cycle progression/proliferation; changes in intracellular metabolism; cell survival	[22]
JAK/STAT	overexpression of cyclin D1; STAT3 dimerization	inhibition of cell apoptosis; nuclear translocation of STAT3	[22]
MAPK	inhibition of MEK1/2; oxidization of p38 cysteine residue	decreased phosphorylation of p38, ERK1/2 and JNK; suppressed activity of p38	[76]

## Abbreviations

ACSL3	Acyl-coenzyme A (CoA) synthetase long-chain family member 3;
AKT	Protein kinase B;
AMPK	5′ AMP-activated protein kinase;
6-AN	6-anicotinamide;
Anlo	Anlotinib;
APC	Adenomatous polyposis coli;
ASK1	Apoptosis signal-regulating kinase 1;
ATPP-DTPA	5-(4-amino-phenyl)-10,15,20-triphenylporphyrin-diethylene-triaminopentaacetic acid;
ATF4	Activating transcription factor 4;
BER	Base excision repair;
BMP	Bone morphogenetic protein;
BRAF	B-Raf proto-oncogene;
CAFs	Cancer-associated fibroblasts;
CAT	Catalase;
CDDP	Cisplatin;
CDK	Cyclin-dependent kinase;
CIN	Chromosomal instability;
CIOSS	CRC-integrated OS score;
CM-H2DCFDA	5-(and 6)-chloromethyl-2′,7′-dichlorohydrofluorescein diacetate;
COX-2	Cyclooxygenase-2;
CPT-11	Irinotecan;
CRC	Colorectal cancer;
CTLA-4	Cytotoxic T lymphocyte-associated protein 4;
CtBP1	C-terminal binding protein-1;
CXCL2	C-X-C motif chemokine ligand 2;
Cyt-c	Cytochrome-c;
DCF	2′,7′-dichlorofluorescein;
DHA	Dihydroartemisinin;
DHE	Dihydroethidium;
d-ROMs	Reactive oxygen metabolite derivatives;
Dvl	Dishevelled protein 1;
DTNB	5,5′-dithio-bis(2-nitrobenzoic acid);
EGFR	Epidermal growth factor receptor;
ELISA	Enzyme-linked immunosorbent assay;
EMT	Epithelial-to-mesenchymal transition;
ER	Endoplasmic reticulum;
ERK	Extracellular signal-regulated kinase;
FGFR	Fibroblast growth factor receptor;
FLIM	Fluorescence lifetime imaging microscopy;
5-FU	5-fluorouracil;
G6PD	Glucose-6-phosphatedehydrogenase;
GGT2	Gamma-glutamyltransferase 2;
GOT1	Glutamic-oxaloacetic transaminase 1;
GPXs	Glutathione peroxidases
GSH	Glutathione;
GSS	Glutathione synthase;
GSSG	Oxidized glutathione
GSTs	Glutathione S-transferases;
Gyp	Gypenosides;
H_2_DCF	2′,7′-dichlorodihydrofluorescein;
H_2_DCFDA	2′,7′-dichlorodihydrofluorescein diacetate;
H_2_O_2_	Hydrogen peroxide;
HER	Human epidermal growth factor receptor;
HIF-1α	Hypoxia-inducible factor 1α;
HPLC	High-pressure liquid chromatography;
HY-PDT	Photodynamic therapy mediated by hypericin;
IBD	Inflammatory bowel diseases;
ICAM-1	Intercellular adhesion molecule 1;
IFN	Interferon;
IFN-α	Interferon-alpha;
IKK	Inhibitor κB kinase;
ISC	Intestinal stem cell;
JAK	Janus kinase;
JNK	c-Jun N-terminal kinase;
Keap1	Kelch-like ECH-associated protein 1;
KRAS	KRAS proto-oncogene, GTPase;
LncRNAs	Long noncoding RNAs;
L-OHP	Oxaliplatin;
mAbs	Monoclonal antibodies;
MAPK	Mitogen-activated protein kinase;
MAP3K	Mitogen-activated protein kinase kinase kinase;
MDA	Malondialdehyde;
MDR	Multidrug resistance;
MEK	Mitogen-activated protein kinase kinase-MAPKK;
MET	Metformin;
MLK3, MAP3K11	Mixed lineage kinase 3;
MMR	Mismatch repair;
MPO	Myeloperoxidase;
MSI	Microsatellite instability;
MSI-H	Microsatellite instability-high;
mTOR	Mammalian target of rapamycin;
MUTYH	MutY homolog Escherichia coli, homolog of MYH, hMYH;
MVs	Membrane vesicles;
NaB	Sodium butyrate;
NAC	N-acety1 cysteine;
NADPH	Nicotinamide adenine dinucleotide phosphate;
NPs	Nanoparticles;
NER	Nucleotide excision repair;
NF-κB	Nuclear factor kappa B;
NIK	NF-κB inducing kinase;
NOTCH	Notch receptor;
NOX	NADPH oxidase;
NRAS	NRAS proto-oncogene, GTPase;
NRF2	Nuclear factor-erythroid 2 p45-related factor 2;
NRX	Nucleoredoxin;
NSAIDs	Nonsteroidal anti-inflammatory drug;
O2•−	Superoxide anion;
OGG1	8-oxoguanine DNA glycosylase 1;
•OH	Hydroxyl radical;
8-OHdG	8-hydroxy-2′-deoxyguanosine;
8-oxoG	8-oxoguanine;
8-oxodG	8-oxo-7, 8-dihydro-2′-deoxyguanosine;
oxLDL	Oxidized low-density lipoprotein;
OS	Oxidative stress
P3C-Asp	Dextran–aspirin nanomedicine;
PD-1	Programmed cell death 1;
PDGF-R	Platelet-derived growth factor receptors;
PDL1	Programmed death ligand 1;
PDT	Photodynamic therapy;
PFL	Positive feedback loop;
P-gp	P-glycoprotein;
PI3K	Phosphatidyl inositol 3-OH kinase;
PKB	Protein kinase B;
PL	Piperlongumine;
PMN	Polymorphonuclear;
PPP	Pentose phosphate pathway;
PRX	Peroxiredoxin;
PTEN	Phosphatase and tensin homolog;
PTKs	Protein tyrosine kinases;
PTP	Protein tyrosine phosphatase;
PUFAs	Polyunsaturated fatty acids;
PL	Piperlongumine;
qPCR	Quantitative real-time polymerase chain reaction;
RAC1	Rac family small GTPase 1;
ROS	Reactive oxygen species;
RTKs	Receptor tyrosine kinases;
SA	Salicylic acid;
SalB	Salvianolic acid B;
SCD1	Stearoyl-CoA desaturase-1;
SLC7A11	Solute carrier family 7 member 11;
SMAD4	SMAD family member 4/mothers against decapentaplegic homolog 4;
SOD	Superoxide dismutase;
STAT	Signal transducer of activators of transcription;
TALDO1	Transaldolase 1;
TAMs	Tumor-associated macrophages;
TANs	Tumor-associated neutrophils;
TBA	Modified 2-thiobarbituric acid;
TBARS	Thiobarbituric acid reactive;
TCA	Tricarboxylic acid cycle;
TCF-4	T-cell factor-4;
TCRs	T-cell receptors;
TIGAR	TP53-induced glycolysis regulatory phosphatase;
TGF-β	Transforming growth factor-β;
TKI	Tyrosine kinase inhibitor;
TME	Tissue microenvironment;
TNFR	Tumor necrosis factor receptor;
TRAF3	Tumor necrosis factor receptor-associated factor 3;
Trx/TrxR	Thioredoxin/thioredoxin reductase;
VCAM1	Vascular cell adhesion molecule 1;
VEGF	Vascular endothelial growth factor;
VEGFR	Vascular endothelial growth factor receptor;
Wnt	Wingless/It;
Wnt-PCP	Wnt-planar cell polarity;
xCT	xc− transporter;
X-PDT	X-ray-induced photodynamic therapy.
